# Proactive vs. reactive car driving: EEG evidence for different driving strategies of older drivers

**DOI:** 10.1371/journal.pone.0191500

**Published:** 2018-01-19

**Authors:** Melanie Karthaus, Edmund Wascher, Stephan Getzmann

**Affiliations:** Department of Ergonomics, IfADo - Leibniz Research Centre for Working Environment and Human Factors at TU Dortmund, Dortmund, Germany; University of Electronic Science and Technology of China, CHINA

## Abstract

Aging is associated with a large heterogeneity in the extent of age-related changes in sensory, motor, and cognitive functions. All these functions can influence the performance in complex tasks like car driving. The present study aims to identify potential differences in underlying cognitive processes that may explain inter-individual variability in driving performance. Younger and older participants performed a one-hour monotonous driving task in a driving simulator under varying crosswind conditions, while behavioral and electrophysiological data were recorded. Overall, younger and older drivers showed comparable driving performance (lane keeping). However, there was a large difference in driving lane variability within the older group. Dividing the older group in two subgroups with low vs. high driving lane variability revealed differences between the two groups in electrophysiological correlates of mental workload, consumption of mental resources, and activation and sustaining of attention: Older drivers with high driving lane variability showed higher frontal Alpha and Theta activity than older drivers with low driving lane variability and—with increasing crosswind—a more pronounced decrease in Beta activity. These results suggest differences in driving strategies of older and younger drivers, with the older drivers using either a rather proactive and alert driving strategy (indicated by low driving lane variability and lower Alpha and Beta activity), or a rather reactive strategy (indicated by high driving lane variability and higher Alpha activity).

## Introduction

Aging is associated with changes in perceptual, motor, and cognitive functioning [1]. Even in healthy aging these changes may have an impact on everyday behavior, especially on complex tasks like driving a car in dense traffic environments [2]. According to Anstey et al. (2012) [3], vision and cognitive factors explain up to 83–95% of age-related variance in driving ability. Cognitive factors comprise slowing in response speed [4], problems in dividing and switching of attention [5, 6], declines of performance in dual- or multitask situations [7], and deficits in inhibition of irrelevant stimuli and of inappropriate responses in the driving context [2, 8]. These impairments are even enhanced under time pressure or in very complex and unpredictable situations [9]. Turning to one’s left, driving on busy roads, and crossing intersections are typical examples of such difficult driving situations. Accident analysis and statistics from different countries confirm these results: Most crashes caused by elderly drivers are the consequences of ignoring the right of way (especially at intersections), and of incorrect (left) turns and lane changes [10, 11].

In addition to a general age-related decline, aging is also associated with an increase in inter-individual variability in cognitive performance [12]. This increase is based—at least in part—on the use of different strategies for compensating age-related declines in functioning, as supposed by the so-called decline-compensation hypothesis [13]. Effective compensation strategies may therefore explain why—despite a general trend of an age-related decline—some studies did not find any differences in driving performance between younger and older drivers [14, 15]. In fact, there is a large variance within the group of older drivers, and only a small percentage of them shows an increased risk of accidents. Many, but not all of those drivers have deficits in basic vision (e.g. due to eye diseases [16]) or preclinical or early stage of dementia [17]. The risk of accidents is also increased in drivers, who are at least 75 years old, and who drive less than 3000 km per year [18]. However, the vast majority of elderly are safe drivers [19], who show no noticeable driving problems, neither in real life, nor in experimental driving simulator settings.

On the other hand, subtle differences in driving competence and driving strategies between older drivers may not become manifest in overt performance. A potential method for exploring the underlying cognitive processes of more or less successful car driving are neurophysiological measures. The electroencephalogram (EEG) allows the exploration of human information processing and cognitive functioning at a very high time resolution in situations, where no overt performance can be measured. One well-established method is the analysis of oscillatory brain activity, with the frequency bands of the EEG reflecting different mental processes of perception and cognition: In the present context, three frequency bands are of particular interest, the Alpha, Theta, and Beta bands. Oscillations in Alpha band (8–13 Hz) are most evident at posterior regions of the head, and are traditionally associated with mental fatigue [20]. However, some studies showed a decrease of Alpha activity with increasing task complexity [21]. It has therefore been supposed that posterior Alpha activity reflects a mental state of boredom or withdrawal of attention, rather than mental fatigue [22, 23]. The frontal Theta activity (4–7 Hz) is associated with mental activity and cognitive control, for example, in reinforcement learning tasks (e.g. [24]; for a review see [25]). Theta activity increases continuously in long-lasting monotonous tasks [26, 27], and therefore seems to reflect the consumption of mental resources with time on task. Finally, Beta activity (> 13 Hz), traditionally associated with sensorimotor functions (for review, see [28]), has also been related to cognitive processing and mental workload [29]. Taken together, the pattern of activity in these frequency bands reflect different mental states that, in turn, may result in different behavioral outcomes.

The present study used these brain oscillatory measures to explore the cortical basis of inter-individual differences in driving performance and driving strategies of younger and older drivers. A one-hour monotonous driving scenario was used, in which the participants had to keep a vehicle on the lane and to countersteer, when crosswind (of different strength) came up to move the car off the road [23]. The underlying mechanisms of high driving abilities were studied by post-hoc subdividing the group of older participants into a high-workload (Old-High) and a low-workload (Old-Low) group. The individual mental workload of compensating crosswind was operationalized by the variability of driving lane, as proposed by Verwey and Veltman (1996) [30]. The subdivision into an Old-High and Old-Low group was based on the assumption that a higher variability of driving lane (indicated by a higher steering activity) should reflect a higher effort of crosswind compensation and, in turn, higher driving workload. A study with young drivers indicated large inter-individual differences in driving lane variability [31], and pilot studies revealed that this was especially true for older drivers, suggesting differences in the amount of mental workload in demanding driving situations.

To investigate the neural correlates of driving workload, the oscillatory activities of the Old-High and Old-Low groups were contrasted: Significant differences in Alpha, Beta, or Theta power would indicate differences in mental fatigue, consumption of mental resources, and mental workload, respectively. In the theoretical framework of a driving task, Garcia et al. (2017) [32] recently proposed two different driving states, a proactive state in which the brain anticipates and actively plans the responses to sensory driving information, and a rather reactive state in which the brain reacts to environmental information. The proactive state is characterized by a strong activity in the Beta and Delta bands, while the reactive state is characterized by activity primarily within the alpha band. In the present study, it was therefore hypothesized that a proactive driving state should be associated with a low driving lane variability in combination with a strong Beta and fronto-central Theta activity in the low-workload group. In contrast, a reactive driving state should be associated with a high driving lane variability and a strong posterior Alpha activity in the high-workload group. Finally, age-related differences in driving workload were explored by comparing the Old-Low group with the group of younger participants (Young). Here it was expected that low-workload older drivers use different (potentially compensating) driving strategies than younger ones. Such compensation in driving strategies should be reflected by additional brain activity in Theta and/or Beta band in the Old-Low group relative to the young group.

## Materials and methods

### Participants

All subjects provided informed written consent and all experimental procedures were approved by the local ethics committee of the Leibniz Research Centre for Working Environment and Human Factors. The sample consisted of 14 younger (mean age = 25.1, SD = 2.7; range 20–31 years; 7 women) and 28 older participants (mean age = 64.6, SD = 3.7; range 56–70 years; 12 women). The younger participants (driving license since 6 years on average) were mainly recruited from local colleges and universities, and the older participants (driving license since 45 years on average) were recruited by regional newspaper advertisements. All participants were experienced drivers, using a car at least twice a week in the last three years. They reported no history of any neurological or psychiatric disorder and no consumption of substances that may affect the central nervous system. All of them had normal or corrected to normal vision and hearing and did not show any signs of simulator sickness during testing. They provided informed written consent prior to entering the experiment and received up to 30 € for their participation. The study complied with the tenets of the Declaration of Helsinki.

### Procedure and stimuli

**Fig 1:**
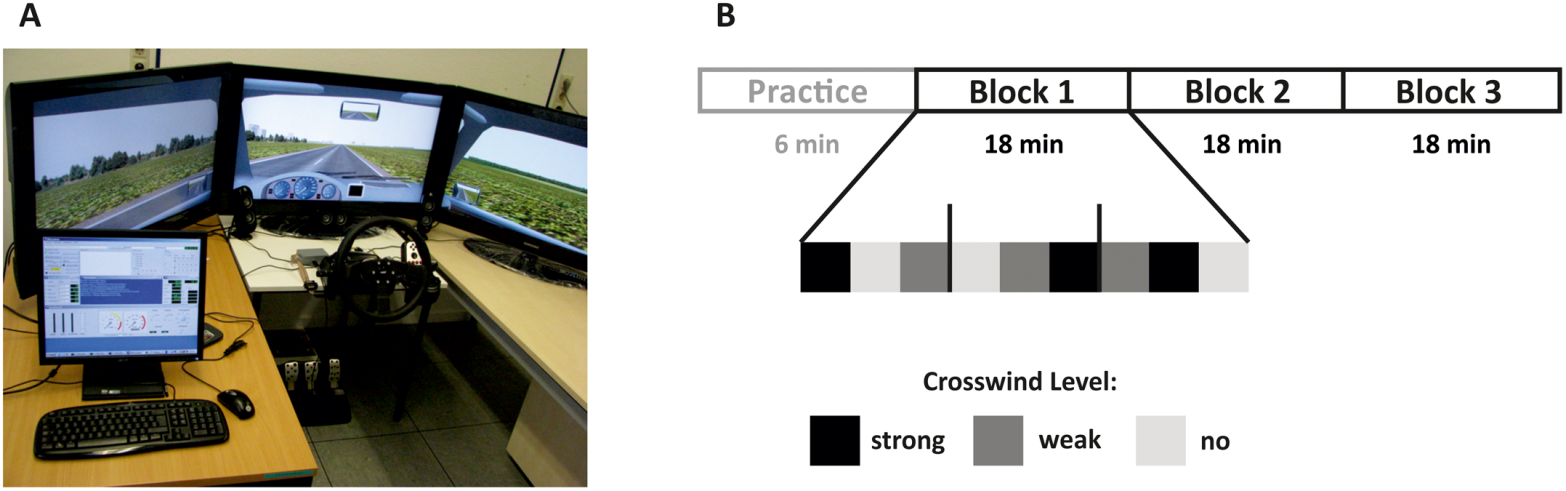
Experimental design. (A) Experimental environment with driving simulator configuration and (B) task set-up with one initial practice block followed by three experimental blocks. Each experimental block consisted of nine segments with three different crosswind levels.

The experiment took place in a static driving simulator (ST Sim; ST Software B.V. Groningen, NL; Fig 1A). The participants had to drive at a constant speed of 31 mph on a monotonous straight two-lane road through grassland. There were no bends or any other visual distraction on the road.

While driving, the participants had to stay accurately with the vehicle on the right lane. To simulate weak and strong crosswind, the slope of the road varied as a function of lateral force of several sine waves (1/25.6, 1/17, 1/12.8, 1/10.2, 1/8.6, 1/7.2, 1/6.4, and 1/5.6 Hz). The sine waves consisted of eight different superimposing and phase-delaying sinusoid signals, and the resulting lateral force was unpredictable for the participants. There were three different crosswind levels (no, weak, and strong with the amplitude of the strong crosswind being twice that of weak crosswind). The crosswind varied every two minutes. Before each 2-minutes segment short transfer-intervals (duration 1 sec) were introduced to avoid artificial changes in crosswind level. During the experiment auditory stimuli consisting of harmonic tones of different frequency (duration 100 ms, interstimulus-onset interval 1000 ms, intensity 65 dB) were presented. The cortical responses to these tone stimuli were the topic of a different study that was not relevant here. The participants could therefore ignore the tones. The experiment started with a practice block in which the participants became familiar with the task. Then three experimental blocks each with nine segments had to be performed (see Fig 1B). The driving task was performed without any break or interruption and lasted 60 minutes.

### Data recording

While driving, the EEG (biosemi active system, Active two, BioSemi, NL) was recorded from 64 scalp electrode sites. EEG electrodes were arranged on the basis of the International 10–10 system and two additional electrodes were placed on the left and right mastoids. The Biosemi’s Active Two amplifier uses a 2-wire active electrode system with a Common Mode Sensing and Driven Right Leg (CMS/DRL) principle. Data were sampled at 2048 Hz and a bandwidth of DC– 140 Hz. Additionally, six electro-oculography (EOG) electrodes were positioned around the two eyes to record horizontal and vertical eye movements. Electrode impedance was kept below 10 kΩ. The current position of the vehicle was continuously recorded by the EEG system.

### Data analysis

#### Behavioral data

Drivers usually have different preferences of the “ideal” car positioning on the road. Therefore, the ideal path was defined for each participant individually on the basis of his/her own driving data. The median of the distance between car and road side averaged across the complete driving road was thereto defined. Based on this individual “ideal” path, the driving error was operationalized as the accuracy of lane keeping and computed as the root-mean-squared deviance from the ideal path of each participant. In addition to the driving error as a measure of lane keeping performance, the driving lane variability was analyzed as a measure of mental workload. The driving lane variability was computed as the standard deviation of the individual path. Both measures, driving error and driving lane variability, were calculated separately for the three levels of crosswind. For analysis of different driving strategies, the group of older participants was subdivided into two subgroups with low vs. high driving lane variability (averaged across all levels of crosswind) by split-half-median, resulting in high-workload (Old-High: high driving lane variability) and low-workload (Old-Low: low driving lane variability) subgroups. In addition, driving lane variability was determined for the entirety of the younger group. While the younger group (mean age 25.1, SD 2.7 years; mean driving years 6.0, SD 2.3) differed significantly in age and years of driving licence from the Old-High group (mean age 65.4, SD 2.2 years; *t*(26) = 43.6; *p* < .001; mean driving years 44.5, SD 3.7 *t*(26) = 33.3; *p* < .001) and the Old-Low group (mean age 63.9, SD 4.8 years; *t*(26) = 26.7; *p* < .001; mean driving years 45.2, SD 3.9; *t*(25) = 31.8; *p* < .001), the two older subgroups did not differ (mean age: *t*(26) = 1.0; *p* > .05; mean driving years: *t*(25) = 0.5; *p* > .05; bonferroni-corrected *t*-tests). Both driving error and driving lane variability were subjected to two-way analyses of variance (ANOVAs) with within-subject factor crosswind condition (no, weak, strong) and between-subject factor group (Old-High, Old-Low, Young). Importantly, there were two separate analyses, with the between-subject factor either being workload or age: One ANOVA tested the factor workload within the group of older drivers (contrasting Old-Low vs. Old-High drivers), and the other one tested the factor age within a group of drivers with comparable low driving lane variability (contrasting Old-Low vs. Young drivers). Levene’s test was used to control the homogeneity of variance and in case of inhomogeneous variances, degrees of freedom were adjusted. To evaluate the practical significance of the findings more accurately, effect sizes (here: partial η^2^) were computed.

#### EEG data

Recorded data were re-referenced using the reference electrode standardization technique (REST, [33]) across all 64 scalp electrodes. REST has some advantages over the commonly used average reference and tends to obtain more accurate and objective results (e.g. [34, 35, 36]). Data were bandpass-filtered (0.5–45 Hz), down-sampled to 128 Hz, and segments from 500 to 1000 ms around the irrelevant auditory distracters were extracted. The period of 200 ms before each tone was used as baseline. Segments with EEG artifacts were removed using the statistics based tools as implemented in EEGLAB. On the cleaned data, an independent component analysis (ICA) was applied. With the aid of ADJUST [37] and additional visual inspection artifacts were semi-automatically identified and removed.

As the spectral properties of the EEG vary strongly between individuals, in particular when different age groups are considered, the frequency bands for each subject were adjusted individually based on the individual alpha frequency (IAF). One method to determine the IAF is the gravity frequency (GF) method. GF is a measure of a central tendency within a given frequency range (most common in the alpha band) and is defined as the weighted sum of spectral estimates, divided by the total power [21, 38]. Based on the GF_**α**_ the theta-range as IAF– 5 Hz to IAF– 3 Hz, the alpha-range as IAF– 2 Hz to IAF + 2 Hz, and the beta range as IAF + 4 to IAF + 18 Hz were defined. Mean power values for Alpha, Beta, and Theta bands were calculated for a fronto-central (FCz) and a posterior electrode site (POz), and subjected to three-way ANOVAs with within-subject factors crosswind condition (no, weak, strong) and electrode site (FCz, POz) and between-subject factor group (contrasting Old-Low vs. Old-High drivers or Old-Low vs. Young drivers; see above).

## Results

### Behavioral data

**Fig 2:**
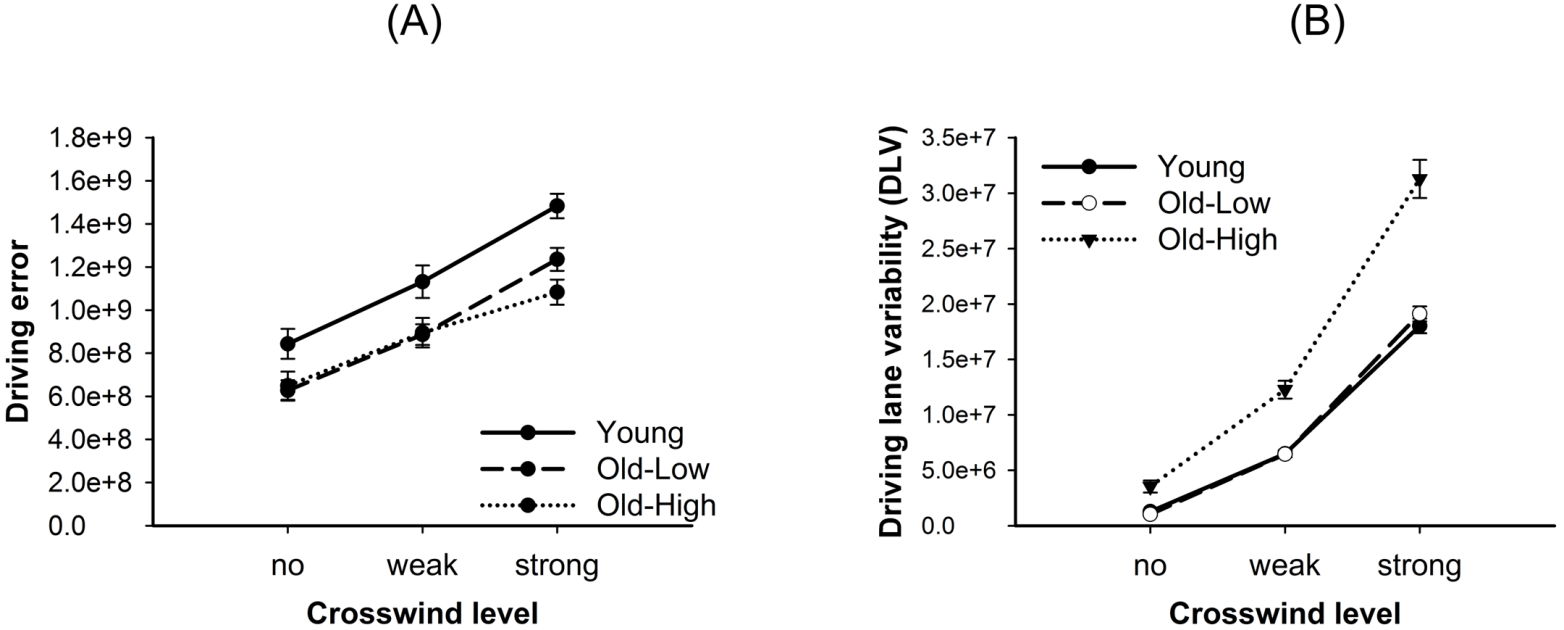
Results of behavioral data. (A) Driving error and (B) driving lane variability as function of crosswind level (no, weak, strong), shown for young participants and older participants with high (Old-High) and low (Old-Low) driving lane variability. Error bars are standard errors.

The driving error increased with increasing crosswind (*F*(2,78) = 67.35, *p* < .001, *η*^*2*^ = .63; Fig 2A). However, the three groups did not differ, neither in overall driving error, nor in the effect of crosswind on driving error (both *p* > .05; *η*^*2*^ ≤ .08). As expected, driving lane variability differed between groups (*F*(2,39) = 9.54, *p* < .001, *η*^*2*^ = .33), and post-hoc *t*-tests confirmed that the Old-High group had a higher driving lane variability than the Old-Low group (*p* = .004), while the Old-Low group did not differ from the Young group (*p* = .768). The differences between groups became even greater with increasing crosswind (*F*(4,78) = 8.56, *p* < .001, *η*^*2*^ = .31; Fig 2B).

### EEG data

#### Young vs. Old-Low group

**Fig 3:**
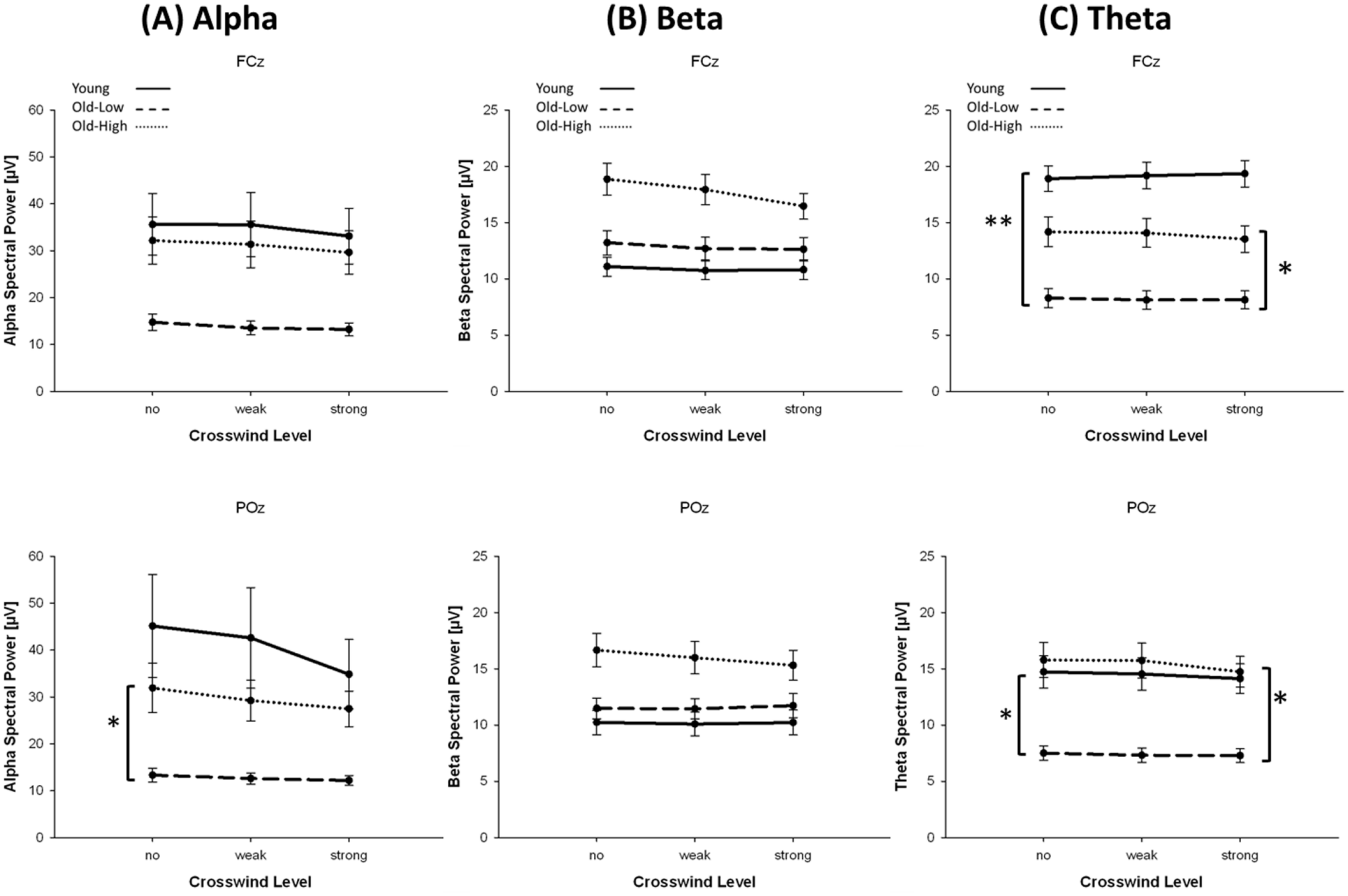
Oscillatory brain activity in different frequency bands. Spectral power (means and standard errors of means) of fronto-central and posterior Alpha (A), (overall) Beta (B) and Theta (C) band as function of crosswind level (no, weak, strong), shown for younger participants and older participants with high (Old-High) and low (Old-Low) driving lane variability. Significant group differences are indicated by asterisks; *p < .05; **p < .01.

The posterior Alpha power did not depend on crosswind level. Also, there was no main effect of group and no interaction of crosswind level and group (all *p* > .05; all *η*^*2*^ < .11; Fig 3A). However, the fronto-central Alpha power was slightly stronger in the Young than Old-Low group (*F*(1,26) = 3.50, *p* = .073, *η*^*2*^ = .12) and decreased with increasing crosswind (*F*(2,52) = 4.43, *p* = .024, *η*^*2*^ = .15). The posterior and fronto-central Theta power was stronger in the Young than Old-Low group (POz: *F*(1,26) = 7.04, *p* = .013, *η*^*2*^ = .21; FCz: *F*(1,26) = 19.34, *p* < .001, *η*^*2*^ = .43), while there were no significant main effects of crosswind level, and no interactions of crosswind level and group (all *p* > .05; all *η*^*2*^ < .08; see Fig 3C). The fronto-central Beta power decreased with increasing crosswind (*F*(2,52) = 4.89, *p* = .020, *η*^*2*^ = .16), while there were no significant main effects of group or interactions of crosswind level and group, neither for posterior, nor fronto-central Beta power (all *p* > .05; all *η*^*2*^ < .03; Fig 3B).

#### Old-High vs. Old-Low

Posterior Alpha power was stronger in the Old-High than Old-Low group (*F*(1,26) = 4.34, *p* = .047, *η*^*2*^ = .14), and there was no main effect of crosswind level and no interaction of group and crosswind level (both *p* > .05; all *η*^*2*^ < .11; Fig 3A). Fronto-central Alpha power was also slightly stronger in the Old-High than Old-Low group (*F*(1,26) = 3.66, *p* = .067, *η*^*2*^ = .12) and decreased with increasing crosswind (*F*(2,52) = 7.81, *p* = .003, *η*^*2*^ = .23), but there was no interaction of crosswind level and group (*p* > .05; *η*^*2*^ < .04). Fronto-central and posterior Theta power decreased with increasing crosswind (FCz: *F*(2,52) = 3.79, *p* = .048, *η*^*2*^ = .13; POz: *F*(2,52) = 3.79, *p* = .055, *η*^*2*^ = .13; Fig 3C). In addition, the Old-High group had a stronger Theta power than the Old-Low group (FCz: *F*(1,26) = 4.68, *p* = .040, *η*^*2*^ = .15; POz: *F*(1,26) = 8.11, *p* = .008, *η*^*2*^ = .24), without any interaction of crosswind and group (both *p* > .05; *η*^*2*^ < .08). Beta Power decreased with increasing crosswind at the fronto-central position (*F*(2,52) = 18.28, *p* < .001, *η*^*2*^ = .41) and—to a lesser degree—posterior position (*F*(2,52) = 3.07, *p* = .074, *η*^*2*^ = .11). While there were no statistically significant main effects of group (both *p* > .05; *η*^*2*^ < .10), there were significant interactions of crosswind level and group (POz: *F*(2,52) = 6.27, *p* = .009, *η*^*2*^ = .19; FCz: *F*(2,52) = 7.41, *p* = .005, *η*^*2*^ = .22; Fig 3B), indicating a greater effect of crosswind (i.e., decreasing Beta power with increasing crosswind) in the Old-High than Old-Low group.

## Discussion

In a one-hour driving simulator lane-keeping task on a monotonous road with different crosswind levels no significant differences in driving error were found between the groups tested. Thus, younger participants and older participants with low and high driving lane variability did not differ in their ability to keep lane on the individual ideal path. Crosswind level had an effect on lane keeping performance: Increasing crosswind, reflecting increasing task difficulty, resulted in larger driving errors in all groups, as also found in previous studies [39, 40]. As a second measure, the driving lane variability was assessed that has been proposed as a correlate of the amount of mental workload mobilized while performing the driving task [30]. A higher variability of driving lane should reflect a higher effort of crosswind compensation and thus higher mental workload. For exploring electrophysiological correlates of mental workload the group of older participants was subdivided into two subgroups with low vs. high driving lane variability. As could be expected, differences in driving lane variability between these two older groups increased with increasing crosswind level, while driving lane variability did not differ between the Old-Low and Young groups.

The analysis of brain oscillations indicated a lower frontal Theta activity of the Old-Low group relative to the Young group. This difference in Theta power could be based on a general age-related decline in frontal Theta activity, which has also been observed in previous studies [41]. Kardos and colleagues (2014) [41] related this decline to the “inability to efficiently recruit attentional resources” of older adults which, for example, may result in deteriorated memory performance. However, given that the Old-Low and Young groups did not differ in driving error and driving lane variability, this age-related decline in frontal Theta activity obviously did not affect driving performance in the present lane-keeping task. In contrast, Old-High participants showed a higher frontal Theta activity than Old-Low participants. Assuming frontal Theta activity to represent cognitive control and mental workload, this suggests that older drivers with high driving lane variability (need to) use more cognitive control to perform the driving task than older drivers with low driving lane variability, possibly resulting in greater demands of attentional resources and higher mental workload. Interestingly, higher frontal Theta activity in the Old-High group came along with higher Alpha activity over posterior and—to a lesser degree—frontal areas (relative to the Old-Low group). High Alpha activity is usually associated with a relaxed mental state [42], drowsiness [43], or some kind of attentional withdrawal [23]. All these mental states may decelerate or reduce the responsiveness to stimuli [44] and enhance the probability of errors, which may be reflected in high driving lane variability in the present task. In contrast, older participants with low driving variability seem to be able to respond to different crosswind levels in a more anticipatory way, possibly due to a generally higher alertness, as indicated by lower Alpha activity.

While the Young and Old-Low groups did not differ, frontal Beta activity was slightly stronger in the Old-High than Old-Low group. Even more important, there was a highly significant interaction of group and crosswind level in Beta activity, indicating that the older participants with high driving lane variability showed a more pronounced decrease of Beta activity with increasing crosswind level than the Old-Low group. Since decreased Beta activity is usually associated with lower mental workload, this result appears to be counterintuitive at the first glance, because older drivers with high driving lane variability tended to show higher mental workload while driving (as indicated by a higher frontal Theta power than the Old-low group). In fact, there are several possible explanations: On the one hand, Beta activity might be associated with attentional modulation rather than with mental workload [45]. In their experiment, Gola and colleagues (2013) [45] adjusted the task difficulty in a way that the behavioral performances of younger and older participants were similar. They found that a subgroup of older participants (“high performers”) did not differ in Beta activity from young participants, whereas another subgroup (“low performers”) showed decreased Beta power in conditions with high task difficulty. This decrease of Beta power has been interpreted to reflect “the difficulty in activation and deficits in sustaining attentional processes” ([45] p. 334). The more prominent decrease in Beta activity with increasing task difficulty that was observed in the Old-High subgroup of the present study suggests that older participants with high driving lane variability had more difficulties in activation and sustaining of attentional processes while driving (as indicated by higher frontal Beta activity) than older drivers with low driving lane variability. These difficulties could be associated with attentional withdrawal (as indicated by high Alpha activity) and the requirement of high cognitive control and mental workload (as indicated by high Theta activity) to adequately perform the driving task.

Alternatively, the decline of Beta activity could be based on a higher oscillatory modulation and/or a stronger desynchronization of EEG power [46]. Hanslmayr and colleagues (2012) [46] demonstrated that desynchronization in alpha and beta power is associated with the encoding and retrieval of memory. Accordingly, referring to mathematical models of information theory, the degree of encoded information is related to the amount of desynchronization, in a way that "… the more information needs to be encoded, the more desynchronized the firing of local neural assemblies needs to be" ([46] p.7). Thus, the relative decrease of Beta power at the high crosswind level (which was most pronounced in the older group with high driving lane variability) may be based on a stronger desynchronization of Beta power, reflecting the increased encoding of information in the most demanding task condition.

The present results are in accordance with the idea of two different neuro-behavioral states that have recently been demonstrated as fluctuations in the on-going oscillatory activity in a driving task with younger participants [32]. The authors distinguished between a *proactive* state in which sensory driving information is anticipated and actively used to plan future responses (characterized by a strong Beta/Delta Activity), and a *reactive* state in which the brain reacts to environmental information (characterized by activity within the alpha band). With reference to the present lane-keeping task, the two subgroups of older drivers could prefer different driving strategies. Drivers of the Old-Low group seem to prefer a rather alert and proactive driving strategy: For keeping the lane as precisely as possible, these drivers kept high attention to compensate for crosswind, as reflected in low driving lane variability. This driving strategy is associated with an overall reduced Alpha and Theta activity. On the other hand, the Old-High drivers responded rather reactive on crosswind, which resulted in a delayed compensatory steering activity and a higher driving lane variability. Thus, more cognitive control was necessary to achieve comparable results in lane keeping. The latter driving strategy was associated with higher consumption of mental resources, as indicated by high frontal Theta activity.

### Conclusion

The present results suggest differences in driving strategies of older and younger drivers, with the older drivers using either a rather proactive and alert driving strategy (indicated by low driving lane variability and lower Alpha and Beta activity), or a rather reactive strategy (indicated by high driving lane variability and higher Alpha activity). As a consequence, the reactive driving strategy might be critical in complex or unpredictable traffic situations, in which extra mental resources are needed to react fast and correctly. Training interventions for improving the traffic safety of older drivers should therefore favour a more proactive and alert driving strategy that should leave more mental resources available for responding to additional critical events while driving.

## Supporting information

S1 Fig Experimental design. (A) Experimental environment with driving simulator configuration and (B) task set-up with one initial practice block followed by three experimental blocks. Each experimental block consisted of nine segments with three different crosswind levels.
(DOCX)

S2 Fig Results of behavioral data. (A) Driving error and (B) driving lane variability as function of crosswind level (no, weak, strong), shown for young participants and older participants with high (Old-High) and low (Old-Low) driving lane variability. Error bars are standard errors.
(DOCX)

S3 Fig Oscillatory brain activity in different frequency bands. Spectral power (means and standard errors of means) of fronto-central and posterior Alpha (A), Beta (B) and Theta (C) band as function of crosswind level (no, weak, strong), shown for younger participants and older participants with high (Old-High) and low (Old-Low) driving lane variability. Significant group differences are indicated by asterisks; *p < .05; **p < .01.
(DOCX)

S1 File Data. Behavioral data.
(SAV)

S2 File Data REST. EEG data based on the reference electrode standardization technique (REST).
(PDF)
