# Supplementary material for: Proactive vs. reactive car driving: EEG evidence for different driving strategies of older drivers
Source: PLoS One. 2018 Jan 19;13(1):e0191500. doi: 10.1371/journal.pone.0191500 (PMC5774811; doi:10.1371/journal.pone.0191500)
Supplement: S1 Fig — (A) Experimental environment with driving simulator configuration and (B) task set-up with one initial practice block followed by three experimental blocks. Each experimental block consisted of nine segments with three different crosswind levels. (DOCX) [file pone.0191500.s001.docx]

**Supporting Information Fig.1**

**S1 Fig. Experimental Design.** (A) Experimental environment with driving simulator configuration and (B) task set-up with one initial practice block followed by three experimental blocks. Each experimental block consisted of nine segments with three different crosswind levels.
