# Supplementary material for: Proactive vs. reactive car driving: EEG evidence for different driving strategies of older drivers
Source: PLoS One. 2018 Jan 19;13(1):e0191500. doi: 10.1371/journal.pone.0191500 (PMC5774811; doi:10.1371/journal.pone.0191500)
Supplement: S2 Fig — (A) Driving error and (B) driving lane variability as function of crosswind level (no, weak, strong), shown for young participants and older participants with high (Old-High) and low (Old-Low) driving lane variability. Error bars are standard errors. (DOCX) [file pone.0191500.s002.docx]

**Supporting Information Fig.2**

**S2 Fig. Results of behavioral data.** (A) Driving error and (B) driving lane variability as function of crosswind level (no, weak, strong), shown for young participants and older participants with high (Old-High) and low (Old-Low) driving lane variability. Error bars are standard errors.
