# Supplementary material for: Proactive vs. reactive car driving: EEG evidence for different driving strategies of older drivers
Source: PLoS One. 2018 Jan 19;13(1):e0191500. doi: 10.1371/journal.pone.0191500 (PMC5774811; doi:10.1371/journal.pone.0191500)
Supplement: S3 Fig — Spectral power (means and standard errors of means) of fronto-central and posterior Alpha (A), Beta (B) and Theta (C) band as function of crosswind level (no, weak, strong), shown for younger participants and older participants with high (Old-High) and low (Old-Low) driving lane variability. Significant group differences are indicated by asterisks; *p < .05; **p < .01. (DOCX) [file pone.0191500.s003.docx]

**Supporting Information Fig.3**

**S3 Fig. Oscillatory brain activity in different frequency bands.** Spectral power (means and standard errors of means) of fronto-central and posterior Alpha (A), Beta (B) and Theta (C) band as function of crosswind level (no, weak, strong), shown for younger participants and older participants with high (Old-High) and low (Old-Low) driving lane variability. Significant group differences are indicated by asterisks; *p < .05; **p < .01.
