# Supplementary material for: Proactive vs. reactive car driving: EEG evidence for different driving strategies of older drivers
Source: PLoS One. 2018 Jan 19;13(1):e0191500. doi: 10.1371/journal.pone.0191500 (PMC5774811; doi:10.1371/journal.pone.0191500)
Supplement: S2 File — EEG data based on the reference electrode standardization technique (REST). (PDF) [file pone.0191500.s005.pdf]

	Alpha Power [µV]	Theta Power [µV]	Beta Power [µV]	Channel	Wind	Block	Subject	Group
1	11.919	13.006	6.3329	FCz	no	1	1	Young
2	9.6973	9.6146	4.8998	POz	no	1	1	Young
3	11.691	12.991	6.5021	FCz	weak	1	1	Young
4	9.032	9.8694	5.4284	POz	weak	1	1	Young
5	12.484	14.879	6.7989	FCz	strong	1	1	Young
6	9.9876	11.354	5.8689	POz	strong	1	1	Young
7	12.511	11.351	4.9055	FCz	no	2	1	Young
8	10.955	9.158	3.9445	POz	no	2	1	Young
9	13.469	14.212	5.2075	FCz	weak	2	1	Young
10	11.802	10.928	4.3158	POz	weak	2	1	Young
11	11.676	13.016	5.2955	FCz	strong	2	1	Young
12	9.539	10.136	4.4159	POz	strong	2	1	Young
13	16.919	16.314	5.2984	FCz	no	3	1	Young
14	15.091	13.123	4.2125	POz	no	3	1	Young
15	14.196	14.505	4.9727	FCz	weak	3	1	Young
16	12.519	13.221	4.2519	POz	weak	3	1	Young
17	15.571	17.9	4.7781	FCz	strong	3	1	Young
18	12.942	13.64	4.4574	POz	strong	3	1	Young
19	28.184	25.821	14.841	FCz	no	1	2	Young
20	9.9901	8.2959	7.0501	POz	no	1	2	Young
21	30.308	29.193	14.234	FCz	weak	1	2	Young
22	9.6367	7.8233	6.6008	POz	weak	1	2	Young
23	30.197	30.712	15.858	FCz	strong	1	2	Young
24	9.2374	7.7611	7.3919	POz	strong	1	2	Young
25	34.241	27.424	15.647	FCz	no	2	2	Young
26	12.404	8.6141	6.2489	POz	no	2	2	Young
27	28.808	26.832	13.662	FCz	weak	2	2	Young
28	10.401	8.1415	5.7839	POz	weak	2	2	Young
29	29.435	27.665	13.942	FCz	strong	2	2	Young
30	9.9304	8.1388	6.2572	POz	strong	2	2	Young
31	37.952	25.779	17.539	FCz	no	3	2	Young
32	14.584	8.9965	6.5762	POz	no	3	2	Young
33	34.466	25.994	16.548	FCz	weak	3	2	Young
34	12.148	7.6678	6.2126	POz	weak	3	2	Young
35	33.44	27.232	14.876	FCz	strong	3	2	Young
36	11.45	8.3475	6.0143	POz	strong	3	2	Young
37	11.433	9.8833	6.2351	FCz	no	1	3	Young
38	11.14	6.3596	4.1973	POz	no	1	3	Young
39	11.817	11.497	6.5679	FCz	weak	1	3	Young
40	11.339	5.9571	4.2605	POz	weak	1	3	Young
41	10.484	11.119	6.5293	FCz	strong	1	3	Young

42	8.8196	6.4835	4.3765	POz	strong	1	3	Young
43	13.53	10.506	7.1117	FCz	no	2	3	Young
44	14.132	5.8525	4.1719	POz	no	2	3	Young
45	13.517	10.934	6.7243	FCz	weak	2	3	Young
46	11.065	6.0615	4.0947	POz	weak	2	3	Young
47	12.403	10.597	6.3595	FCz	strong	2	3	Young
48	10.005	6.0532	3.9948	POz	strong	2	3	Young
49	13.412	11.246	6.6378	FCz	no	3	3	Young
50	12.222	6.6814	3.7974	POz	no	3	3	Young
51	13.506	10.865	6.9986	FCz	weak	3	3	Young
52	9.8644	5.945	4.0593	POz	weak	3	3	Young
53	12.935	9.9909	6.8374	FCz	strong	3	3	Young
54	10.7	5.6738	4.1015	POz	strong	3	3	Young
55	104.63	23.202	17.827	FCz	no	1	4	Young
56	180.29	30.174	24.771	POz	no	1	4	Young
57	104.6	21.353	17.425	FCz	weak	1	4	Young
58	135.73	26.825	23.798	POz	weak	1	4	Young
59	89.888	23.269	18.359	FCz	strong	1	4	Young
60	116.93	26.525	24.78	POz	strong	1	4	Young
61	184.84	25.547	24.007	FCz	no	2	4	Young
62	262.06	41.289	25.841	POz	no	2	4	Young
63	187.25	31.849	21.984	FCz	weak	2	4	Young
64	263.75	36.051	24.821	POz	weak	2	4	Young
65	165.88	25.352	21.332	FCz	strong	2	4	Young
66	187.03	32.549	23.558	POz	strong	2	4	Young
67	189.16	24.352	19.744	FCz	no	3	4	Young
68	334.51	33.08	24.511	POz	no	3	4	Young
69	205.11	31.135	21.95	FCz	weak	3	4	Young
70	321.06	36.631	22.71	POz	weak	3	4	Young
71	175.32	27.897	21.834	FCz	strong	3	4	Young
72	206.83	31.026	22.036	POz	strong	3	4	Young
73	38.106	24.772	13.487	FCz	no	1	5	Young
74	71.425	27.467	15.747	POz	no	1	5	Young
75	41.662	22.976	12.493	FCz	weak	1	5	Young
76	63.641	24.96	14.602	POz	weak	1	5	Young
77	54.205	25.309	13.028	FCz	strong	1	5	Young
78	78.479	28.48	14.959	POz	strong	1	5	Young
79	116.09	26.897	17.25	FCz	no	2	5	Young
80	188.57	33.664	19.535	POz	no	2	5	Young
81	111.64	26.572	16.655	FCz	weak	2	5	Young
82	218.34	35.733	20.424	POz	weak	2	5	Young
83	94.204	28.374	16.378	FCz	strong	2	5	Young
84	142.46	29.838	17.038	POz	strong	2	5	Young
85	74.996	24.527	15.084	FCz	no	3	5	Young
86	137.32	26.739	16.959	POz	no	3	5	Young

87	86.613	25.703	15.427	FCz	weak	3	5	Young
88	114.63	26.796	15.224	POz	weak	3	5	Young
89	84.977	25.175	15.198	FCz	strong	3	5	Young
90	108.79	28.426	14.851	POz	strong	3	5	Young
91	19.237	25.404	7.3163	FCz	no	1	6	Young
92	21.434	15.085	5.1916	POz	no	1	6	Young
93	21.451	24.667	7.3987	FCz	weak	1	6	Young
94	20.859	14.037	4.898	POz	weak	1	6	Young
95	22.006	23.325	6.6747	FCz	strong	1	6	Young
96	19.068	15.368	4.6869	POz	strong	1	6	Young
97	20.843	22.401	6.9986	FCz	no	2	6	Young
98	19.554	12.48	4.9609	POz	no	2	6	Young
99	21.314	21.867	7.6721	FCz	weak	2	6	Young
100	19.454	12.774	5.4372	POz	weak	2	6	Young
101	19.338	20.291	7.1474	FCz	strong	2	6	Young
102	19.7	12.743	4.8775	POz	strong	2	6	Young
103	20.626	21.313	7.5546	FCz	no	3	6	Young
104	20.123	12.946	4.7711	POz	no	3	6	Young
105	20.728	21.612	6.7858	FCz	weak	3	6	Young
106	18.907	12.533	4.569	POz	weak	3	6	Young
107	18.957	20.659	6.7943	FCz	strong	3	6	Young
108	17.423	12.48	4.6702	POz	strong	3	6	Young
109	12.234	22.836	8.1899	FCz	no	1	7	Young
110	23.108	21.894	12.209	POz	no	1	7	Young
111	13.64	25.651	8.6428	FCz	weak	1	7	Young
112	20.068	21.963	12.585	POz	weak	1	7	Young
113	12.819	25.706	8.2781	FCz	strong	1	7	Young
114	17.981	21.238	12.181	POz	strong	1	7	Young
115	13.731	23.546	8.8078	FCz	no	2	7	Young
116	25.894	19.664	11.915	POz	no	2	7	Young
117	14.326	24.432	9.3949	FCz	weak	2	7	Young
118	24.087	21.138	12.179	POz	weak	2	7	Young
119	15.25	24.962	8.3937	FCz	strong	2	7	Young
120	21.122	21.568	11.39	POz	strong	2	7	Young
121	14.916	22.819	9.5286	FCz	no	3	7	Young
122	24.201	22.326	11.905	POz	no	3	7	Young
123	14.668	24.741	8.6867	FCz	weak	3	7	Young
124	22.429	21.218	11.711	POz	weak	3	7	Young
125	14.251	24.027	8.6405	FCz	strong	3	7	Young
126	20.493	19.406	11.224	POz	strong	3	7	Young
127	8.4663	7.3133	4.919	FCz	no	1	8	Young
128	3.4014	2.6713	2.1328	POz	no	1	8	Young
129	7.5742	7.3935	4.9224	FCz	weak	1	8	Young
130	3.0049	2.6543	2.1681	POz	weak	1	8	Young
131	7.7497	6.6636	4.6014	FCz	strong	1	8	Young

132	3.3432	2.6162	2.1407	POz	strong	1	8	Young
133	8.2541	6.3688	4.3124	FCz	no	2	8	Young
134	3.0196	2.4369	1.8298	POz	no	2	8	Young
135	7.8741	5.9757	4.0319	FCz	weak	2	8	Young
136	2.7742	2.4105	1.8249	POz	weak	2	8	Young
137	7.3243	6.2154	4.0802	FCz	strong	2	8	Young
138	2.9824	2.3837	1.8257	POz	strong	2	8	Young
139	8.1799	6.7037	4.1786	FCz	no	3	8	Young
140	3.1514	2.2531	1.7755	POz	no	3	8	Young
141	6.477	5.3516	3.587	FCz	weak	3	8	Young
142	2.7171	2.2122	1.6753	POz	weak	3	8	Young
143	7.2554	6.1647	3.7486	FCz	strong	3	8	Young
144	3.5119	2.4632	1.7868	POz	strong	3	8	Young
145	15.985	15.321	7.5336	FCz	no	1	9	Young
146	10.379	7.9401	4.3184	POz	no	1	9	Young
147	16.86	15.24	7.3009	FCz	weak	1	9	Young
148	10.386	7.9477	4.3166	POz	weak	1	9	Young
149	16.011	16.407	6.9271	FCz	strong	1	9	Young
150	10.143	8.1925	3.8177	POz	strong	1	9	Young
151	17.724	15.898	8.8315	FCz	no	2	9	Young
152	11.458	8.3326	4.9779	POz	no	2	9	Young
153	20.282	16.083	8.2513	FCz	weak	2	9	Young
154	11.117	8.2617	4.571	POz	weak	2	9	Young
155	17.773	16.171	7.9974	FCz	strong	2	9	Young
156	9.8185	7.868	4.7689	POz	strong	2	9	Young
157	17.456	17.251	9.5301	FCz	no	3	9	Young
158	9.4281	9.4372	5.4412	POz	no	3	9	Young
159	19.213	15.622	8.7174	FCz	weak	3	9	Young
160	10.333	8.7356	4.9661	POz	weak	3	9	Young
161	18.217	14.792	8.1059	FCz	strong	3	9	Young
162	9.4012	7.5252	4.4953	POz	strong	3	9	Young
163	68.521	25.41	18.242	FCz	no	1	10	Young
164	60.543	18.264	15.216	POz	no	1	10	Young
165	67.86	26.777	18.451	FCz	weak	1	10	Young
166	61.209	16.401	18.484	POz	weak	1	10	Young
167	55.293	27.907	19.749	FCz	strong	1	10	Young
168	41.689	15.126	20.613	POz	strong	1	10	Young
169	63.775	26.045	22.581	FCz	no	2	10	Young
170	48.601	19.753	21.73	POz	no	2	10	Young
171	59.326	26.869	22.205	FCz	weak	2	10	Young
172	48.888	16.697	23.213	POz	weak	2	10	Young
173	58.328	27.107	22.912	FCz	strong	2	10	Young
174	49.282	16.065	23.565	POz	strong	2	10	Young
175	68.527	29.68	23.4	FCz	no	3	10	Young
176	59.928	17.087	23.878	POz	no	3	10	Young

177	65.502	25.708	22.13	FCz	weak	3	10	Young
178	53.081	16.389	21.463	POz	weak	3	10	Young
179	66.763	25.656	24.162	FCz	strong	3	10	Young
180	54.842	16.583	25.175	POz	strong	3	10	Young
181	18.61	14.914	8.1422	FCz	no	1	11	Young
182	15.318	9.6256	9.417	POz	no	1	11	Young
183	13.58	13.207	7.6638	FCz	weak	1	11	Young
184	11.568	9.5844	9.5407	POz	weak	1	11	Young
185	12.833	15.138	7.4801	FCz	strong	1	11	Young
186	10.347	8.8504	8.8528	POz	strong	1	11	Young
187	23.299	16.876	11.534	FCz	no	2	11	Young
188	17.265	11.773	10.696	POz	no	2	11	Young
189	21.35	15.288	10.27	FCz	weak	2	11	Young
190	13.974	9.7511	10.34	POz	weak	2	11	Young
191	17.643	14.42	9.4572	FCz	strong	2	11	Young
192	14.826	9.8358	9.583	POz	strong	2	11	Young
193	32.724	18.635	13.949	FCz	no	3	11	Young
194	20.927	12.056	12.412	POz	no	3	11	Young
195	24.22	17.31	13.249	FCz	weak	3	11	Young
196	16.303	12.303	11.352	POz	weak	3	11	Young
197	22.916	17.033	12.725	FCz	strong	3	11	Young
198	16.042	11.704	11.655	POz	strong	3	11	Young
199	27.546	30.441	14.179	FCz	no	1	12	Young
200	27.935	24.661	17.129	POz	no	1	12	Young
201	25.885	29	13.595	FCz	weak	1	12	Young
202	27.316	26.562	17.653	POz	weak	1	12	Young
203	26.422	30.504	15.086	FCz	strong	1	12	Young
204	27.334	23.481	18.278	POz	strong	1	12	Young
205	26.732	28.17	15.799	FCz	no	2	12	Young
206	26.702	24.036	18.641	POz	no	2	12	Young
207	26.514	27.624	13.732	FCz	weak	2	12	Young
208	27.735	25.419	16.945	POz	weak	2	12	Young
209	29.29	29.294	17.439	FCz	strong	2	12	Young
210	29.703	25.876	20.84	POz	strong	2	12	Young
211	24.958	26.839	12.41	FCz	no	3	12	Young
212	25.162	21.116	15.89	POz	no	3	12	Young
213	26.971	27.858	13.013	FCz	weak	3	12	Young
214	29.07	26.969	16.895	POz	weak	3	12	Young
215	27.307	29.009	14.159	FCz	strong	3	12	Young
216	29.382	24.211	18.728	POz	strong	3	12	Young
217	14.029	9.5521	8.7641	FCz	no	1	13	Young
218	34.919	8.9638	6.0117	POz	no	1	13	Young
219	11.948	8.6588	7.9487	FCz	weak	1	13	Young
220	26.884	8.8965	5.8365	POz	weak	1	13	Young
221	10.242	9.3852	6.6443	FCz	strong	1	13	Young

222	20.505	8.6752	5.1513	POz	strong	1	13	Young
223	14.502	8.4566	8.229	FCz	no	2	13	Young
224	31.763	9.1446	5.8758	POz	no	2	13	Young
225	13.599	8.5245	7.4907	FCz	weak	2	13	Young
226	31.464	8.5524	5.5552	POz	weak	2	13	Young
227	11.509	9.1306	7.267	FCz	strong	2	13	Young
228	26.842	8.7568	5.3846	POz	strong	2	13	Young
229	17.307	9.1704	8.0025	FCz	no	3	13	Young
230	38.58	9.7098	5.8175	POz	no	3	13	Young
231	14.921	9.9369	7.5082	FCz	weak	3	13	Young
232	33.233	9.4186	6.0217	POz	weak	3	13	Young
233	13.871	8.9858	6.661	FCz	strong	3	13	Young
234	26.688	9.6477	5.5165	POz	strong	3	13	Young
235	9.1551	14.756	6.2777	FCz	no	1	14	Young
236	8.4144	8.985	6.9174	POz	no	1	14	Young
237	9.3403	15.008	6.6221	FCz	weak	1	14	Young
238	8.6541	8.9119	7.1242	POz	weak	1	14	Young
239	8.6724	15.012	6.487	FCz	strong	1	14	Young
240	8.6464	8.2275	7.126	POz	strong	1	14	Young
241	8.8413	12.82	6.5089	FCz	no	2	14	Young
242	8.7373	7.9085	7.0394	POz	no	2	14	Young
243	8.7811	13.919	6.3252	FCz	weak	2	14	Young
244	8.1286	8.0572	6.8881	POz	weak	2	14	Young
245	8.6011	14.141	6.7761	FCz	strong	2	14	Young
246	8.9484	8.734	7.3178	POz	strong	2	14	Young
247	9.912	13.877	7.9384	FCz	no	3	14	Young
248	10.737	8.6379	9.176	POz	no	3	14	Young
249	10.315	14.096	8.0288	FCz	weak	3	14	Young
250	9.7964	7.8528	8.8638	POz	weak	3	14	Young
251	10.274	15.666	8.02	FCz	strong	3	14	Young
252	9.6282	9.1992	9.4591	POz	strong	3	14	Young
253	22.818	12.278	12.876	FCz	no	1	15	Old-Low
254	14.539	8.7766	9.5864	POz	no	1	15	Old-Low
255	20.86	13.585	10.987	FCz	weak	1	15	Old-Low
256	13.807	9.318	9.2638	POz	weak	1	15	Old-Low
257	21.273	12.558	11.843	FCz	strong	1	15	Old-Low
258	14.488	8.9833	9.3617	POz	strong	1	15	Old-Low
259	27.223	15.119	14.208	FCz	no	2	15	Old-Low
260	18.141	12.294	10.436	POz	no	2	15	Old-Low
261	27.684	13.983	12.211	FCz	weak	2	15	Old-Low
262	18.071	10.063	8.8834	POz	weak	2	15	Old-Low
263	24.743	11.978	10.811	FCz	strong	2	15	Old-Low
264	16.355	9.5823	8.6241	POz	strong	2	15	Old-Low
265	28.819	14.663	15.027	FCz	no	3	15	Old-Low
266	20.105	11.115	10.556	POz	no	3	15	Old-Low

267	29.229	13.385	13.334	FCz	weak	3	15	Old-Low
268	19.246	10.362	10.214	POz	weak	3	15	Old-Low
269	25.372	12.868	12.479	FCz	strong	3	15	Old-Low
270	16.495	9.7666	9.2449	POz	strong	3	15	Old-Low
271	4.1839	2.2467	12.021	FCz	no	1	16	Old-Low
272	4.6039	3.1213	11.092	POz	no	1	16	Old-Low
273	3.8403	2.3444	13.012	FCz	weak	1	16	Old-Low
274	4.3293	3.1842	11.96	POz	weak	1	16	Old-Low
275	4.4607	2.5117	14.703	FCz	strong	1	16	Old-Low
276	5.1023	3.2686	13.824	POz	strong	1	16	Old-Low
277	4.2103	2.4081	10.359	FCz	no	2	16	Old-Low
278	5.2917	3.3317	10.456	POz	no	2	16	Old-Low
279	4.0673	2.3794	9.4325	FCz	weak	2	16	Old-Low
280	5.0489	3.4191	9.7755	POz	weak	2	16	Old-Low
281	4.0238	2.2492	11.332	FCz	strong	2	16	Old-Low
282	5.1577	3.3855	11.52	POz	strong	2	16	Old-Low
283	4.7894	2.2668	8.7512	FCz	no	3	16	Old-Low
284	5.8061	3.5367	9.6349	POz	no	3	16	Old-Low
285	4.9164	2.4916	9.7122	FCz	weak	3	16	Old-Low
286	5.8339	3.7941	11.111	POz	weak	3	16	Old-Low
287	3.7746	2.1346	7.116	FCz	strong	3	16	Old-Low
288	4.4585	3.2735	7.7441	POz	strong	3	16	Old-Low
289	7.9975	5.49	10.182	FCz	no	1	17	Old-Low
290	6.6867	3.8411	9.6729	POz	no	1	17	Old-Low
291	8.1443	5.4913	10.686	FCz	weak	1	17	Old-Low
292	7.4852	4.1442	11.25	POz	weak	1	17	Old-Low
293	8.8	5.5177	10.76	FCz	strong	1	17	Old-Low
294	7.6141	4.2185	11.346	POz	strong	1	17	Old-Low
295	8.529	6.2918	9.2218	FCz	no	2	17	Old-Low
296	6.6158	4.1185	7.8788	POz	no	2	17	Old-Low
297	8.5363	5.232	9.455	FCz	weak	2	17	Old-Low
298	6.9069	3.4878	8.9798	POz	weak	2	17	Old-Low
299	8.3172	5.5493	8.3166	FCz	strong	2	17	Old-Low
300	6.7599	3.5783	7.7505	POz	strong	2	17	Old-Low
301	8.567	5.4026	7.4003	FCz	no	3	17	Old-Low
302	6.7533	3.5747	5.0101	POz	no	3	17	Old-Low
303	7.9835	5.3475	8.1162	FCz	weak	3	17	Old-Low
304	6.6229	3.4076	6.6162	POz	weak	3	17	Old-Low
305	8.3381	4.881	6.3741	FCz	strong	3	17	Old-Low
306	6.4798	3.3095	4.9573	POz	strong	3	17	Old-Low
307	5.0099	3.9889	8.1085	FCz	no	1	18	Old-Low
308	5.3826	7.1235	6.7143	POz	no	1	18	Old-Low
309	5.3403	4.2668	8.752	FCz	weak	1	18	Old-Low
310	5.4521	6.4427	7.791	POz	weak	1	18	Old-Low
311	6.2349	4.4334	8.3736	FCz	strong	1	18	Old-Low

312	7.083	7.9426	7.4418	POz	strong	1	18	Old-Low
313	5.8829	3.8295	7.4739	FCz	no	2	18	Old-Low
314	5.49	5.9819	6.8579	POz	no	2	18	Old-Low
315	5.1367	3.9743	7.8864	FCz	weak	2	18	Old-Low
316	5.0164	6.3886	7.7656	POz	weak	2	18	Old-Low
317	5.617	4.2449	7.7003	FCz	strong	2	18	Old-Low
318	6.2644	7.5455	7.538	POz	strong	2	18	Old-Low
319	6.4859	3.9962	8.5015	FCz	no	3	18	Old-Low
320	5.9197	5.4575	8.3005	POz	no	3	18	Old-Low
321	6.2302	4.0689	7.749	FCz	weak	3	18	Old-Low
322	5.8534	5.9343	7.3571	POz	weak	3	18	Old-Low
323	6.1739	4.2731	7.6999	FCz	strong	3	18	Old-Low
324	6.09	6.6187	7.6948	POz	strong	3	18	Old-Low
325	8.078	3.6965	7.4917	FCz	no	1	19	Old-Low
326	10.629	4.9236	6.205	POz	no	1	19	Old-Low
327	7.9457	3.9269	6.4785	FCz	weak	1	19	Old-Low
328	10.28	4.6671	5.4736	POz	weak	1	19	Old-Low
329	7.3817	3.8014	5.4396	FCz	strong	1	19	Old-Low
330	9.0372	5.3108	5.2285	POz	strong	1	19	Old-Low
331	9.527	4.2433	7.6972	FCz	no	2	19	Old-Low
332	12.82	5.0163	6.1657	POz	no	2	19	Old-Low
333	9.0412	4.2791	6.7427	FCz	weak	2	19	Old-Low
334	12.154	5.4273	5.7396	POz	weak	2	19	Old-Low
335	8.3109	3.9183	6.0293	FCz	strong	2	19	Old-Low
336	10.619	5.4751	5.0741	POz	strong	2	19	Old-Low
337	10.331	4.0034	7.4189	FCz	no	3	19	Old-Low
338	13.984	5.1662	6.2692	POz	no	3	19	Old-Low
339	10.069	3.9103	7.288	FCz	weak	3	19	Old-Low
340	14.352	5.318	6.1814	POz	weak	3	19	Old-Low
341	8.0943	3.5364	6.432	FCz	strong	3	19	Old-Low
342	10.637	4.8511	5.4543	POz	strong	3	19	Old-Low
343	42.364	22.284	21.057	FCz	no	1	20	Old-Low
344	36.699	12.79	8.5774	POz	no	1	20	Old-Low
345	34.486	20.751	19.369	FCz	weak	1	20	Old-Low
346	27.912	11.041	7.7818	POz	weak	1	20	Old-Low
347	32.938	21.929	18.566	FCz	strong	1	20	Old-Low
348	24.88	10.824	7.5069	POz	strong	1	20	Old-Low
349	46.575	22.007	22.092	FCz	no	2	20	Old-Low
350	39.717	13.138	8.3466	POz	no	2	20	Old-Low
351	42.069	22.49	20.963	FCz	weak	2	20	Old-Low
352	35.913	13.477	8.2598	POz	weak	2	20	Old-Low
353	36.511	22.164	18.955	FCz	strong	2	20	Old-Low
354	30.049	11.894	7.6841	POz	strong	2	20	Old-Low
355	55.249	25.443	22.849	FCz	no	3	20	Old-Low
356	45.915	14.422	8.8971	POz	no	3	20	Old-Low

357	38.752	23.055	20.752	FCz	weak	3	20	Old-Low
358	34.792	13.338	8.3834	POz	weak	3	20	Old-Low
359	40.464	23.188	20.674	FCz	strong	3	20	Old-Low
360	30.431	12.069	7.6667	POz	strong	3	20	Old-Low
361	10.21	6.5936	9.3001	FCz	no	1	21	Old-Low
362	8.004	3.7589	7.3092	POz	no	1	21	Old-Low
363	9.7435	5.7328	9.663	FCz	weak	1	21	Old-Low
364	8.0362	3.3687	7.4693	POz	weak	1	21	Old-Low
365	9.7374	6.0882	9.0217	FCz	strong	1	21	Old-Low
366	8.042	3.4182	7.0167	POz	strong	1	21	Old-Low
367	12.167	6.3306	11.189	FCz	no	2	21	Old-Low
368	9.0643	3.9635	8.0242	POz	no	2	21	Old-Low
369	9.9749	6.0296	10.374	FCz	weak	2	21	Old-Low
370	7.7098	3.5263	7.2056	POz	weak	2	21	Old-Low
371	10.643	6.0598	9.3681	FCz	strong	2	21	Old-Low
372	8.5428	3.6542	6.9492	POz	strong	2	21	Old-Low
373	12.282	6.1224	10.691	FCz	no	3	21	Old-Low
374	8.502	3.655	7.7387	POz	no	3	21	Old-Low
375	10.612	6.1511	10.516	FCz	weak	3	21	Old-Low
376	8.0773	3.4702	7.2914	POz	weak	3	21	Old-Low
377	11.498	6.435	10.265	FCz	strong	3	21	Old-Low
378	8.4203	3.4478	7.447	POz	strong	3	21	Old-Low
379	10.403	8.2354	14.565	FCz	no	1	22	Old-Low
380	10.566	7.3458	19.417	POz	no	1	22	Old-Low
381	9.4082	8.3089	13.777	FCz	weak	1	22	Old-Low
382	10.434	6.72	17.871	POz	weak	1	22	Old-Low
383	9.9615	8.7089	14.995	FCz	strong	1	22	Old-Low
384	10.24	6.8233	20.39	POz	strong	1	22	Old-Low
385	11.186	8.6396	12.86	FCz	no	2	22	Old-Low
386	10.678	8.1453	14.825	POz	no	2	22	Old-Low
387	10.376	9.0307	12.159	FCz	weak	2	22	Old-Low
388	11.08	7.3871	14.254	POz	weak	2	22	Old-Low
389	10.521	8.2933	13.215	FCz	strong	2	22	Old-Low
390	11.172	6.7827	16.63	POz	strong	2	22	Old-Low
391	11.545	8.723	18.263	FCz	no	3	22	Old-Low
392	13.017	8.7344	21.46	POz	no	3	22	Old-Low
393	11.985	9.302	15.444	FCz	weak	3	22	Old-Low
394	12.201	8.7508	18.333	POz	weak	3	22	Old-Low
395	11.605	8.9737	17.821	FCz	strong	3	22	Old-Low
396	12.439	7.861	21.012	POz	strong	3	22	Old-Low
397	11.593	4.024	20.241	FCz	no	1	23	Old-Low
398	12.103	2.7736	22.152	POz	no	1	23	Old-Low
399	10.285	4.0893	20.586	FCz	weak	1	23	Old-Low
400	12.129	2.8962	23.146	POz	weak	1	23	Old-Low
401	11.998	4.3032	22.332	FCz	strong	1	23	Old-Low

402	14.048	3.5303	26.146	POz	strong	1	23	Old-Low
403	10.363	3.8388	21.596	FCz	no	2	23	Old-Low
404	11.625	2.5609	21.665	POz	no	2	23	Old-Low
405	9.1953	3.7816	18.501	FCz	weak	2	23	Old-Low
406	10.621	2.6444	20.348	POz	weak	2	23	Old-Low
407	9.0333	4.1011	18.854	FCz	strong	2	23	Old-Low
408	10.722	2.9297	21.736	POz	strong	2	23	Old-Low
409	8.1546	3.5637	16.904	FCz	no	3	23	Old-Low
410	7.8916	2.3515	15.056	POz	no	3	23	Old-Low
411	8.6501	3.8023	16.696	FCz	weak	3	23	Old-Low
412	8.0105	2.3599	16.591	POz	weak	3	23	Old-Low
413	7.8002	3.8812	14.145	FCz	strong	3	23	Old-Low
414	7.7621	2.4143	14.097	POz	strong	3	23	Old-Low
415	4.6816	3.4887	6.1157	FCz	no	1	24	Old-Low
416	2.4239	1.4429	4.4285	POz	no	1	24	Old-Low
417	4.0507	3.0837	6.9773	FCz	weak	1	24	Old-Low
418	2.0951	1.3136	5.1159	POz	weak	1	24	Old-Low
419	3.9572	3.174	7.4803	FCz	strong	1	24	Old-Low
420	2.2948	1.3389	5.7238	POz	strong	1	24	Old-Low
421	4.9618	3.3336	6.7737	FCz	no	2	24	Old-Low
422	2.4516	1.3524	4.3756	POz	no	2	24	Old-Low
423	4.5814	3.1807	7.1199	FCz	weak	2	24	Old-Low
424	2.445	1.3099	5.2647	POz	weak	2	24	Old-Low
425	4.3062	3.1707	5.9873	FCz	strong	2	24	Old-Low
426	2.0609	1.359	4.1184	POz	strong	2	24	Old-Low
427	5.5254	3.1995	7.9323	FCz	no	3	24	Old-Low
428	2.8725	1.4442	5.8844	POz	no	3	24	Old-Low
429	6.0639	3.1579	6.297	FCz	weak	3	24	Old-Low
430	2.9975	1.3208	4.4916	POz	weak	3	24	Old-Low
431	5.4174	3.2216	6.8835	FCz	strong	3	24	Old-Low
432	2.6437	1.3925	4.8886	POz	strong	3	24	Old-Low
433	19.541	11.211	30.662	FCz	no	1	25	Old-Low
434	17.354	10.418	22.327	POz	no	1	25	Old-Low
435	20.762	10.972	26	FCz	weak	1	25	Old-Low
436	17.845	9.5806	20.155	POz	weak	1	25	Old-Low
437	20.666	10.822	25.853	FCz	strong	1	25	Old-Low
438	18.053	10.308	21.405	POz	strong	1	25	Old-Low
439	26.599	12.287	33.977	FCz	no	2	25	Old-Low
440	20.804	10.852	25.043	POz	no	2	25	Old-Low
441	23.791	12.12	30.933	FCz	weak	2	25	Old-Low
442	19.141	10.514	22.47	POz	weak	2	25	Old-Low
443	24.694	11.131	31.963	FCz	strong	2	25	Old-Low
444	20.04	10.094	26.96	POz	strong	2	25	Old-Low
445	29.871	15.292	30.314	FCz	no	3	25	Old-Low
446	20.099	11.832	19.687	POz	no	3	25	Old-Low

447	24.252	11.954	33.518	FCz	weak	3	25	Old-Low
448	19.226	9.8332	24.075	POz	weak	3	25	Old-Low
449	23.441	12.383	32.243	FCz	strong	3	25	Old-Low
450	19.197	10.127	24.601	POz	strong	3	25	Old-Low
451	11.913	8.5076	19.238	FCz	no	1	26	Old-Low
452	14.802	11.533	26.243	POz	no	1	26	Old-Low
453	12.353	9.1526	20.01	FCz	weak	1	26	Old-Low
454	15.558	13.951	27.751	POz	weak	1	26	Old-Low
455	13.421	10.501	23.177	FCz	strong	1	26	Old-Low
456	17.856	15.955	31.429	POz	strong	1	26	Old-Low
457	8.6529	7.1848	9.8194	FCz	no	2	26	Old-Low
458	10.818	10.36	13.599	POz	no	2	26	Old-Low
459	8.1593	7.8423	9.3497	FCz	weak	2	26	Old-Low
460	11.238	11.139	13.493	POz	weak	2	26	Old-Low
461	9.0134	8.2062	11.971	FCz	strong	2	26	Old-Low
462	11.966	11.646	16.627	POz	strong	2	26	Old-Low
463	7.5771	6.6277	5.7923	FCz	no	3	26	Old-Low
464	9.6004	10.653	8.5359	POz	no	3	26	Old-Low
465	8.0453	6.3995	7.2842	FCz	weak	3	26	Old-Low
466	10.345	10.746	9.8121	POz	weak	3	26	Old-Low
467	8.0961	6.8232	6.7137	FCz	strong	3	26	Old-Low
468	10.144	11.571	9.4444	POz	strong	3	26	Old-Low
469	11.179	8.755	9.4691	FCz	no	1	27	Old-Low
470	16.167	11.383	10.151	POz	no	1	27	Old-Low
471	14.102	8.2753	9.9829	FCz	weak	1	27	Old-Low
472	17.681	11.799	10.391	POz	weak	1	27	Old-Low
473	12.808	9.0621	9.4399	FCz	strong	1	27	Old-Low
474	16.989	10.569	9.7417	POz	strong	1	27	Old-Low
475	13.1	9.5962	9.202	FCz	no	2	27	Old-Low
476	16.064	12.63	10.186	POz	no	2	27	Old-Low
477	14.354	9.5076	9.2807	FCz	weak	2	27	Old-Low
478	19.259	11.986	10.764	POz	weak	2	27	Old-Low
479	12.59	9.3393	8.5996	FCz	strong	2	27	Old-Low
480	17.482	10.503	9.3703	POz	strong	2	27	Old-Low
481	14.313	9.0993	8.9108	FCz	no	3	27	Old-Low
482	18.325	12.526	9.6079	POz	no	3	27	Old-Low
483	11.977	9.1144	8.0862	FCz	weak	3	27	Old-Low
484	15.445	10.948	9.4906	POz	weak	3	27	Old-Low
485	12.748	9.7121	8.6967	FCz	strong	3	27	Old-Low
486	17.009	10.656	9.3968	POz	strong	3	27	Old-Low
487	18.065	11.228	9.8673	FCz	no	1	28	Old-Low
488	16.853	12.27	12.046	POz	no	1	28	Old-Low
489	16.849	12.406	9.4711	FCz	weak	1	28	Old-Low
490	16.128	13.613	11.483	POz	weak	1	28	Old-Low
491	15.732	11.541	9.95	FCz	strong	1	28	Old-Low

492	14.429	11.563	11.201	POz	strong	1	28	Old-Low
493	19.629	10.604	9.0143	FCz	no	2	28	Old-Low
494	18.114	12.849	10.932	POz	no	2	28	Old-Low
495	15.232	10.955	8.5225	FCz	weak	2	28	Old-Low
496	16.108	12.462	10.498	POz	weak	2	28	Old-Low
497	16.531	11.764	8.6893	FCz	strong	2	28	Old-Low
498	15.575	12.383	10.175	POz	strong	2	28	Old-Low
499	18.006	11.791	9.1955	FCz	no	3	28	Old-Low
500	16.351	12.59	10.666	POz	no	3	28	Old-Low
501	17.529	11.427	8.8875	FCz	weak	3	28	Old-Low
502	15.967	12.366	9.926	POz	weak	3	28	Old-Low
503	17.561	11.801	8.6913	FCz	strong	3	28	Old-Low
504	16.226	13.544	10.007	POz	strong	3	28	Old-Low
505	11.689	8.8414	14.891	FCz	no	1	29	Old-High
506	16.31	8.7832	13.722	POz	no	1	29	Old-High
507	9.1993	7.4176	12.927	FCz	weak	1	29	Old-High
508	12.887	7.2013	13.748	POz	weak	1	29	Old-High
509	8.0515	7.2212	11.279	FCz	strong	1	29	Old-High
510	11.209	7.0831	12.436	POz	strong	1	29	Old-High
511	13.101	7.8698	13.526	FCz	no	2	29	Old-High
512	16.771	7.3625	11.223	POz	no	2	29	Old-High
513	14.561	8.9511	12.626	FCz	weak	2	29	Old-High
514	15.733	8.1637	10.828	POz	weak	2	29	Old-High
515	13.604	7.3583	12.963	FCz	strong	2	29	Old-High
516	18.032	8.3435	11.916	POz	strong	2	29	Old-High
517	19.953	10.206	14.303	FCz	no	3	29	Old-High
518	22.558	9.0939	10.969	POz	no	3	29	Old-High
519	14.125	8.1351	14.669	FCz	weak	3	29	Old-High
520	17.528	9.9012	9.2992	POz	weak	3	29	Old-High
521	17.017	8.6746	12.644	FCz	strong	3	29	Old-High
522	20.262	8.613	9.3723	POz	strong	3	29	Old-High
523	6.13	5.2079	14.069	FCz	no	1	30	Old-High
524	5.64	4.0952	12.979	POz	no	1	30	Old-High
525	5.5286	5.5898	9.45	FCz	weak	1	30	Old-High
526	4.6767	4.0124	8.7868	POz	weak	1	30	Old-High
527	5.104	5.1948	10.849	FCz	strong	1	30	Old-High
528	4.9538	4.1244	11.347	POz	strong	1	30	Old-High
529	6.4194	5.8852	12.377	FCz	no	2	30	Old-High
530	6.1148	4.3005	11.081	POz	no	2	30	Old-High
531	5.6901	5.5635	11.92	FCz	weak	2	30	Old-High
532	5.2046	3.8015	10.86	POz	weak	2	30	Old-High
533	5.5563	5.212	9.6021	FCz	strong	2	30	Old-High
534	5.067	3.6699	10.225	POz	strong	2	30	Old-High
535	6.2418	5.2752	11.903	FCz	no	3	30	Old-High
536	5.7061	3.9978	9.8321	POz	no	3	30	Old-High

537	6.2946	5.5348	14.481	FCz	weak	3	30	Old-High
538	5.7197	3.9834	12.748	POz	weak	3	30	Old-High
539	6.0992	5.4393	14.395	FCz	strong	3	30	Old-High
540	5.8796	3.7777	12.582	POz	strong	3	30	Old-High
541	43.846	13.417	27.393	FCz	no	1	31	Old-High
542	31.696	10.738	21.323	POz	no	1	31	Old-High
543	44.025	13.744	28.632	FCz	weak	1	31	Old-High
544	28.908	10.696	22.114	POz	weak	1	31	Old-High
545	36.753	13.131	24.057	FCz	strong	1	31	Old-High
546	25.405	9.407	19.971	POz	strong	1	31	Old-High
547	55.881	14.943	31.583	FCz	no	2	31	Old-High
548	39.491	11.313	25.558	POz	no	2	31	Old-High
549	50.956	15.029	31.327	FCz	weak	2	31	Old-High
550	36.487	12.228	24.269	POz	weak	2	31	Old-High
551	53.962	15.646	25.868	FCz	strong	2	31	Old-High
552	36.663	12.111	19.507	POz	strong	2	31	Old-High
553	55.714	15.739	30.198	FCz	no	3	31	Old-High
554	38.596	11.702	21.91	POz	no	3	31	Old-High
555	52.76	15.721	26.885	FCz	weak	3	31	Old-High
556	36.511	11.528	19.62	POz	weak	3	31	Old-High
557	48.029	14.831	25.369	FCz	strong	3	31	Old-High
558	34.697	11.556	20.042	POz	strong	3	31	Old-High
559	20.917	9.8357	23.688	FCz	no	1	32	Old-High
560	20.669	15.417	15.662	POz	no	1	32	Old-High
561	18.888	10.232	21.47	FCz	weak	1	32	Old-High
562	20.492	15.381	15.263	POz	weak	1	32	Old-High
563	18.805	10.202	20.128	FCz	strong	1	32	Old-High
564	18.777	12.275	14.504	POz	strong	1	32	Old-High
565	25.338	10.751	27.743	FCz	no	2	32	Old-High
566	23.523	17.665	15.28	POz	no	2	32	Old-High
567	23.522	10.598	24.484	FCz	weak	2	32	Old-High
568	21.298	14.016	13.573	POz	weak	2	32	Old-High
569	21.765	9.7223	20.302	FCz	strong	2	32	Old-High
570	19.286	14.101	12.046	POz	strong	2	32	Old-High
571	22.648	10.206	26.429	FCz	no	3	32	Old-High
572	24.275	15.761	15.896	POz	no	3	32	Old-High
573	23.905	10.467	24.62	FCz	weak	3	32	Old-High
574	21.41	13.178	14.034	POz	weak	3	32	Old-High
575	22.451	9.4443	22.764	FCz	strong	3	32	Old-High
576	21.941	12.705	13.061	POz	strong	3	32	Old-High
577	8.068	13.562	8.2821	FCz	no	1	33	Old-High
578	6.3005	6.8051	6.855	POz	no	1	33	Old-High
579	7.5985	13.302	7.4876	FCz	weak	1	33	Old-High
580	6.6657	7.5437	6.0915	POz	weak	1	33	Old-High
581	7.0491	14.171	7.5183	FCz	strong	1	33	Old-High

582	6.0611	7.0695	6.9356	POz	strong	1	33	Old-High
583	8.5697	13.428	8.2415	FCz	no	2	33	Old-High
584	7.2181	8.6241	6.3274	POz	no	2	33	Old-High
585	8.5513	12.666	7.4047	FCz	weak	2	33	Old-High
586	6.9599	7.7962	5.6378	POz	weak	2	33	Old-High
587	7.6475	12.469	7.1649	FCz	strong	2	33	Old-High
588	6.5226	7.8199	6.1418	POz	strong	2	33	Old-High
589	9.3681	12.992	7.4215	FCz	no	3	33	Old-High
590	7.8928	8.1303	5.2198	POz	no	3	33	Old-High
591	8.6455	13.86	6.7969	FCz	weak	3	33	Old-High
592	7.0941	8.9218	4.7444	POz	weak	3	33	Old-High
593	7.4961	12.349	5.5972	FCz	strong	3	33	Old-High
594	6.0197	8.2414	4.4003	POz	strong	3	33	Old-High
595	87.465	31.023	25.239	FCz	no	1	34	Old-High
596	59.373	20.766	16.634	POz	no	1	34	Old-High
597	88.713	32.801	25.357	FCz	weak	1	34	Old-High
598	57.345	24.311	16.526	POz	weak	1	34	Old-High
599	79.442	29.201	23.133	FCz	strong	1	34	Old-High
600	54.734	20.867	16.15	POz	strong	1	34	Old-High
601	96.631	32.489	26.877	FCz	no	2	34	Old-High
602	65.026	22.592	18.109	POz	no	2	34	Old-High
603	108.24	27.784	24.766	FCz	weak	2	34	Old-High
604	69.173	18.19	17.268	POz	weak	2	34	Old-High
605	98.207	28.577	24.038	FCz	strong	2	34	Old-High
606	64.871	20.789	15.912	POz	strong	2	34	Old-High
607	112.59	33.388	29.779	FCz	no	3	34	Old-High
608	71.996	24.075	20.667	POz	no	3	34	Old-High
609	112.72	32.064	27.803	FCz	weak	3	34	Old-High
610	71.707	23.195	19.382	POz	weak	3	34	Old-High
611	104.53	31.035	24.768	FCz	strong	3	34	Old-High
612	68.02	20.785	17.462	POz	strong	3	34	Old-High
613	103.83	31.772	38.976	FCz	no	1	35	Old-High
614	120.06	30.023	52.636	POz	no	1	35	Old-High
615	93.869	33.699	37.792	FCz	weak	1	35	Old-High
616	103.9	29.299	51.128	POz	weak	1	35	Old-High
617	91.55	29.06	34.993	FCz	strong	1	35	Old-High
618	88.81	24.105	48.692	POz	strong	1	35	Old-High
619	103.81	31.595	30.228	FCz	no	2	35	Old-High
620	161.96	34.577	38.237	POz	no	2	35	Old-High
621	103.16	31.114	27.917	FCz	weak	2	35	Old-High
622	115.25	34.089	36.264	POz	weak	2	35	Old-High
623	80.866	30.696	24.272	FCz	strong	2	35	Old-High
624	91.371	28.314	34.522	POz	strong	2	35	Old-High
625	102.4	34.098	29.911	FCz	no	3	35	Old-High
626	122.13	42.77	40.261	POz	no	3	35	Old-High

627	87.241	30.453	25.067	FCz	weak	3	35	Old-High
628	103.64	37.342	36.997	POz	weak	3	35	Old-High
629	97.095	27.768	23.939	FCz	strong	3	35	Old-High
630	95.475	31.538	37.622	POz	strong	3	35	Old-High
631	32.683	17.297	24.441	FCz	no	1	36	Old-High
632	41.665	34.806	24.454	POz	no	1	36	Old-High
633	34	16.674	25.305	FCz	weak	1	36	Old-High
634	41.388	35.33	24.665	POz	weak	1	36	Old-High
635	33.59	16.637	22.793	FCz	strong	1	36	Old-High
636	43.522	32.173	20.553	POz	strong	1	36	Old-High
637	41.146	19.618	28.25	FCz	no	2	36	Old-High
638	43.62	35.386	26.016	POz	no	2	36	Old-High
639	42.665	19.735	31.515	FCz	weak	2	36	Old-High
640	46.379	36.393	26.871	POz	weak	2	36	Old-High
641	35.683	17.82	25.152	FCz	strong	2	36	Old-High
642	46.247	32.769	22.959	POz	strong	2	36	Old-High
643	52.32	19.009	32.527	FCz	no	3	36	Old-High
644	52.269	32.102	29.28	POz	no	3	36	Old-High
645	45.889	21.653	31.676	FCz	weak	3	36	Old-High
646	50.594	33.764	28.675	POz	weak	3	36	Old-High
647	46.587	17.777	28.39	FCz	strong	3	36	Old-High
648	49.321	32.732	25.754	POz	strong	3	36	Old-High
649	5.8017	7.3303	8.1887	FCz	no	1	37	Old-High
650	8.1305	10.019	6.0763	POz	no	1	37	Old-High
651	5.882	7.7143	8.0141	FCz	weak	1	37	Old-High
652	8.2857	9.6271	6.1071	POz	weak	1	37	Old-High
653	5.8656	7.9666	7.6345	FCz	strong	1	37	Old-High
654	8.3004	9.5905	5.4916	POz	strong	1	37	Old-High
655	5.4434	7.7631	8.1615	FCz	no	2	37	Old-High
656	7.8188	9.6919	5.9717	POz	no	2	37	Old-High
657	5.3039	7.05	7.4974	FCz	weak	2	37	Old-High
658	8.0407	9.2922	5.9822	POz	weak	2	37	Old-High
659	5.4433	6.8219	7.4162	FCz	strong	2	37	Old-High
660	7.7272	9.629	5.8565	POz	strong	2	37	Old-High
661	5.6396	7.8147	8.358	FCz	no	3	37	Old-High
662	7.2025	8.1692	6.3353	POz	no	3	37	Old-High
663	5.5139	7.2128	8.3285	FCz	weak	3	37	Old-High
664	7.0082	8.7691	6.0745	POz	weak	3	37	Old-High
665	5.559	7.1851	8.0996	FCz	strong	3	37	Old-High
666	7.3301	9.109	5.8525	POz	strong	3	37	Old-High
667	11.889	8.8054	8.7898	FCz	no	1	38	Old-High
668	29.803	26.812	14.163	POz	no	1	38	Old-High
669	12.143	9.2085	8.6003	FCz	weak	1	38	Old-High
670	30.902	26.943	14.218	POz	weak	1	38	Old-High
671	11.743	8.8376	9.3901	FCz	strong	1	38	Old-High

672	29.253	27.757	14.782	POz	strong	1	38	Old-High
673	11.9	9.3124	7.46	FCz	no	2	38	Old-High
674	29.885	26.727	12.121	POz	no	2	38	Old-High
675	11.717	9.305	8.1513	FCz	weak	2	38	Old-High
676	27.597	26.615	12.22	POz	weak	2	38	Old-High
677	12.383	8.6302	7.9605	FCz	strong	2	38	Old-High
678	28.091	25.06	12.26	POz	strong	2	38	Old-High
679	12.408	9.7699	7.2512	FCz	no	3	38	Old-High
680	30.657	25.818	11.513	POz	no	3	38	Old-High
681	12.131	9.1941	7.0597	FCz	weak	3	38	Old-High
682	34.333	28.459	11.691	POz	weak	3	38	Old-High
683	12.278	8.7031	7.7101	FCz	strong	3	38	Old-High
684	33.122	27.471	11.902	POz	strong	3	38	Old-High
685	7.9389	8.4914	12.602	FCz	no	1	39	Old-High
686	10.217	13.847	16.167	POz	no	1	39	Old-High
687	8.2762	8.1827	12.199	FCz	weak	1	39	Old-High
688	11.678	15.399	15.735	POz	weak	1	39	Old-High
689	8.8269	9.2573	12.793	FCz	strong	1	39	Old-High
690	10.445	12.955	17.804	POz	strong	1	39	Old-High
691	7.7082	7.5593	12.139	FCz	no	2	39	Old-High
692	8.8118	10.289	14.098	POz	no	2	39	Old-High
693	7.7029	8.7595	12.507	FCz	weak	2	39	Old-High
694	9.2803	9.9839	16.212	POz	weak	2	39	Old-High
695	8.3158	9.2099	13.222	FCz	strong	2	39	Old-High
696	9.7444	11.735	17.525	POz	strong	2	39	Old-High
697	7.2007	7.9531	12.113	FCz	no	3	39	Old-High
698	8.6015	8.8311	14.915	POz	no	3	39	Old-High
699	7.9344	7.8895	11.19	FCz	weak	3	39	Old-High
700	8.4603	9.7193	13.574	POz	weak	3	39	Old-High
701	7.8334	8.149	11.164	FCz	strong	3	39	Old-High
702	9.3769	9.6995	15.362	POz	strong	3	39	Old-High
703	9.4715	5.6816	9.6297	FCz	no	1	40	Old-High
704	8.1792	3.7126	9.0042	POz	no	1	40	Old-High
705	9.4896	5.6464	9.8218	FCz	weak	1	40	Old-High
706	8.8004	3.8464	8.8196	POz	weak	1	40	Old-High
707	8.6158	6.3032	9.0433	FCz	strong	1	40	Old-High
708	7.5694	4.1943	8.6745	POz	strong	1	40	Old-High
709	12.209	5.7839	10.787	FCz	no	2	40	Old-High
710	12.095	4.282	9.8547	POz	no	2	40	Old-High
711	12.071	6.1147	10.483	FCz	weak	2	40	Old-High
712	10.711	4.1159	9.413	POz	weak	2	40	Old-High
713	8.5734	5.699	9.7165	FCz	strong	2	40	Old-High
714	7.3156	3.9645	8.922	POz	strong	2	40	Old-High
715	17.351	7.2505	11.919	FCz	no	3	40	Old-High
716	18.305	4.9213	10.953	POz	no	3	40	Old-High

717	15.667	7.8466	12.21	FCz	weak	3	40	Old-High
718	15.86	5.549	11.682	POz	weak	3	40	Old-High
719	15.901	6.5523	12.357	FCz	strong	3	40	Old-High
720	15.942	4.9877	11.827	POz	strong	3	40	Old-High
721	11.391	13.083	20.59	FCz	no	1	41	Old-High
722	12.417	14.852	14.998	POz	no	1	41	Old-High
723	11.664	12.995	20.065	FCz	weak	1	41	Old-High
724	14.134	16.253	16.091	POz	weak	1	41	Old-High
725	11.704	14.356	17.816	FCz	strong	1	41	Old-High
726	15.23	15.913	14.44	POz	strong	1	41	Old-High
727	12.077	11.868	20.251	FCz	no	2	41	Old-High
728	12.432	14.234	14.131	POz	no	2	41	Old-High
729	12.907	13.114	18.288	FCz	weak	2	41	Old-High
730	13.202	15.033	12.489	POz	weak	2	41	Old-High
731	11.002	13.725	16.423	FCz	strong	2	41	Old-High
732	12.29	14.957	12.043	POz	strong	2	41	Old-High
733	12.955	12.65	22	FCz	no	3	41	Old-High
734	11.685	12.715	14.945	POz	no	3	41	Old-High
735	11.999	12.129	20.456	FCz	weak	3	41	Old-High
736	11.572	14.516	13.605	POz	weak	3	41	Old-High
737	12.193	13.69	17.299	FCz	strong	3	41	Old-High
738	12.62	13.885	12.435	POz	strong	3	41	Old-High
739	44.417	17.624	21.722	FCz	no	1	42	Old-High
740	31.432	15.534	17.178	POz	no	1	42	Old-High
741	51.721	17.546	20.552	FCz	weak	1	42	Old-High
742	33.703	14.409	15.145	POz	weak	1	42	Old-High
743	46.104	17.393	20.394	FCz	strong	1	42	Old-High
744	29.819	14.903	14.138	POz	strong	1	42	Old-High
745	51.249	18.521	24.199	FCz	no	2	42	Old-High
746	39.101	15.906	18.095	POz	no	2	42	Old-High
747	50.808	19.003	20.657	FCz	weak	2	42	Old-High
748	31.907	15.57	15.695	POz	weak	2	42	Old-High
749	47.149	17.171	19.634	FCz	strong	2	42	Old-High
750	29.42	13.399	13.864	POz	strong	2	42	Old-High
751	56.459	19.206	27.354	FCz	no	3	42	Old-High
752	41.798	15.151	18.577	POz	no	3	42	Old-High
753	52.479	19.201	24.07	FCz	weak	3	42	Old-High
754	34.345	16.433	16.877	POz	weak	3	42	Old-High
755	57.341	18.732	21.003	FCz	strong	3	42	Old-High
756	36.451	14.285	15.485	POz	strong	3	42	Old-High
